# The Mirage of Medical Miracles

**DOI:** 10.1007/s10912-025-09942-9

**Published:** 2025-03-26

**Authors:** Yamac Akgun

**Affiliations:** 1 Children’s Hospital of Los Angeles, Los Angeles, CA 90027 USA; 2 University of Southern California, Los Angeles, CA 90007 USA

**Keywords:** Medical miracles, Regenerative medicine, Biotechnology

They spoke of seeds that sprout new flesh,

Of limbs reborn, of hearts refreshed.

A failing lung, a fractured spine,

Would mend itself—just give it time.

They swore they’d shape new life from clay,

Forge flesh anew, keep death at bay.

A future built on steel and light,

Where arteries pulse with borrowed light.

They whispered change in twisted strands,

Rewrite the code with careful hands.

A birth unchained, a fate undone,

Yet still, the errors multiply—one by one.

They bottled youth in crimson streams,

A sip, a shot, eternal dreams.

Yet time, unbowed, just laughed and stayed,

Each wrinkle carved, each hope delayed.

They built machines within our veins,

So small, yet none remain.

They swore they'd weave new lifeblood flows,

But still, we bleed, and still, we die.

For miracle cures, patients wait,

For every dream, the years dictate.

That not all is lost, but all is slow,

The road is long, the trials grow.

And still, we chase, and still, we try,

A wish, a wait, a patient’s sigh.

## Data Availability

No datasets were generated or analysed during the current study.

